# Complete Genome Sequence of Mulberry Vein Banding Associated Virus, a New Tospovirus Infecting Mulberry

**DOI:** 10.1371/journal.pone.0136196

**Published:** 2015-08-20

**Authors:** Jiaorong Meng, Pingping Liu, Liling Zhu, Chengwu Zou, Jieqiu Li, Baoshan Chen

**Affiliations:** 1 College of Agriculture, Guangxi University, Nanning, China; 2 State Key Laboratory for Conservation and Utilization of Subtropical Agro-bioresources (Guangxi University) and Key Laboratory of Ministry of Education of China for Microbial and Plant Genetic Engineering, Nanning, China; Washington State University, UNITED STATES

## Abstract

Mulberry vein banding associated virus (MVBaV) that infects mulberry plants with typical vein banding symptoms had been identified as a tentative species of the genus *Tospovirus* based on the homology of N gene sequence to those of *tospoviruses*. In this study, the complete sequence of the tripartite RNA genome of MVBaV was determined and analyzed. The L RNA has 8905 nucleotides (nt) and encodes the putative RNA-dependent RNA polymerase (RdRp) of 2877 aa amino acids (aa) in the viral complementary (vc) strand. The RdRp of MVBaV shares the highest aa sequence identity (85.9%) with that of *Watermelon silver mottle virus* (WSMoV), and contains conserved motifs shared with those of the species of the genus *Tospovirus*. The M RNA contains 4731 nt and codes in ambisense arrangement for the NSm protein of 309 aa in the sense strand and the Gn/Gc glycoprotein precursor (GP) of 1,124 aa in the vc strand. The NSm and GP of MVBaV share the highest aa sequence identities with those of Capsicum chlorosis virus (CaCV) and *Groundnut bud necrosis virus* (GBNV) (83.2% and 84.3%, respectively). The S RNA is 3294 nt in length and contains two open reading frames (ORFs) in an ambisense coding strategy, encoding a 439-aa non-structural protein (NSs) and the 277-aa nucleocapsid protein (N), respectively. The NSs and N also share the highest aa sequence identity (71.1% and 74.4%, respectively) with those of CaCV. Phylogenetic analysis of the RdRp, NSm, GP, NSs, and N proteins showed that MVBaV is most closely related to CaCV and GBNV and that these proteins cluster with those of the WSMoV serogroup, and that MVBaV seems to be a species bridging the two subgroups within the WSMoV serogroup of tospoviruses in evolutionary aspect, suggesting that MVBaV represents a distinct tospovirus. Analysis of S RNA sequence uncovered the highly conserved 5’-/3’-ends and the coding regions, and the variable region of IGR with divergent patterns among MVBaV isolates.

## Introduction

The mulberry (*Morus spp*.) is an economically important plant grown widely throughout Asia, for the cultivation of the silkworms (*Bombyx mori* Linn.) and for the sericulture industry. Diverse virus-like symptoms, including mosaic, vein banding, vein necrosis, chlorotic ringspots, and leaf deformation were frequently observed on mulberry. The viral diseases have been a major factor restricting yield and quality of mulberry. Two mulberry-infecting viruses, *Mulberry latent virus* (Genus *Carlavirus*) [1] and the *Mulberry ringspot virus* (Genus *Nepovirus*) [2], have been partly characterized in Japan. Recently, a mulberry-infecting *Tospovirus*, temporarily named Mulberry vein banding virus (MuVBV), was found in China and identified to be a new species of *Tospovirus* based on the homology of N protein sequence to other *Tospovirus* species [3].

Tospoviruses cause significant loss of yield and quality to vegetables, legumes, and ornamental crops worldwide and are transmitted by thrips in a circulative and propagative manner [4, 5]. Typical symptoms induced by members of the *Tospovirus* genus include foliar necrotic spots, necrotic stems, bronzing, wilting, and ring spots in leaves and fruits [4, 6]. Tospoviruses are characterized with enveloped quasi-spherical particles of approximately 80–120 nm in size and a tripartite negative and ambisense RNA genome, i.e. small RNA (S RNA), medium RNA (M RMA), and large RNA (L RNA) [4]. The 3' termini of all RNA segments are highly conserved in the first 9 nucleotides and show inverted complementarity to the 5' ends [7].

The L RNA is with a negative polarity and encodes a putative RNA-dependent RNA polymerase (RdRp), which is also called L protein, in the viral complementary (vc) strand for virus replication [4]. The other two genomic RNAs use an ambisense coding strategy. The M RNA encodes a cell-to-cell movement protein (NSm) and the envelope glycoprotein precursor (GP), whereas the S RNA encodes a nonstructural RNA-silencing suppressor protein (NSs) and the nucleocapsid protein (N) [7, 8]. The open reading frames (ORFs) in the M and S RNA segments are separated by large AU-rich intergenic regions (IGR), which forms a stable hairpin structure and is assumed to be involved in transcription termination [4, 9].

In recent years, new tospoviruses such as Pepper necrotic spot virus (PNSV) [10], Soybean vein necrosis associated virus (SVNaV) [11], Hippeastratum chlorotic ring virus (HCRV) [12–14], Bean necrotic mosaic virus (BeNMV) [15], Tomato necrotic spot virus (TNSV) [16], MuVBV [3], and Lisianthus necrotic ringspot virus (LNRV) [17] have been identified.

Tospoviruses are classified into four major serogroups, designated by their serological relatedness to the type viruses *Watermelon silver mottle virus* (WSMoV), *Tomato spotted wilt virus* (TSWV), *Groundnut yellow spot virus* (GYSV), and Iris yellow spot virus (IYSV) [18,19]. *Impatiens necrotic spot virus* (INSV) was classified together as a distinct serotype [18]. BeNMV and SVNaV, phylogenetically clustered into a new branch and exhibited low cross-reactivity with other species, may represent a new evolutionary lineage within the genus *Tospovirus* [15, 20]. As of to date, the full genome sequences were available for sixteen species in the genus *Tospovirus* [8, 11, 13, 21–24].

In our previous survey, a new tospovirus tentatively named Mulberry vein banding virus (MuVBV) with highest N protein homology of 74.4% to Capsicum chlorosis virus (CaCV) was identified from mulberry with vein-banding symptom in Guangxi Province of China by RT-PCR and sequencing of a fragment of the S RNA of the viral genome [3]. Since this virus has not been fully characterized, particularly due to the lack of the vector information, we rename this virus Mulberry vein banding associated virus (MVBaV), to more precisely reflect its current taxonomic status. To better understand the nature of this virus, we cloned and sequenced the whole genome of MVBaV, and characterized its histopathology and serology properties. Our results show that MVBaV is a distinct member of *Tospovirus* belonging to the WSMoV serogroup.

## Material and Methods

### Ethics Statement

This work was supported by the government of Guangxi province and the sampling activities were conducted with the permission of the agricultural authority of local counties or authorities in charged. The mulberry orchards where the plant samples were collected were in public domain and in regular agricultural farm lands where no endangered or protected species were involved. The sampling locations of the isolates of MVBaV in this study and the authorities who issued the permission were listed in S1 Table.

### Virus sources, electron microscopy and indirect enyzme-linked immunosorbent assay (ELISA)

During 2011 to 2012, ten isolates of MVBaV designated XCSY-3, XCBY-1, XZDL-1, SL-3, HX-2, NN-5, NN-10, NN-16, YZ-3, YZ-4, were collected from mulberry plants showing characteristic symptoms including vein banding, mosaic, chlorotic ringspots, necrotic ringspots or vein necrosis on leaves in mulberry orchards around Guangxi province, China. Due to difficulty of sap transmission, the virus was maintained on mulberry by grafting to the virus-free mulberry seedlings. The leaf samples were stored at −80°C until total RNA was extracted. Ultrathin sections of diseased tissues were prepared as described [25] and examined with the H-7650 transmission electron microscope (Hitachi High-Technologies, Tokyo, Japan).

Mixed antisera of WSMoV/*Groundnut bud necrosis virus* (GBNV) for WSMoV serogroup, *Groundnut ringspot virus* (GRSV)/*Tomato chlorotic spot virus* (TCSV) for TSWV serotype, and antisera against TSWV for TSWV serogroup, IYSV for IYSV serogroup, and INSV for the distinct serotype, were purchased from Agdia Inc. (Elkhart, IN, USA). Indirect ELISA was performed as described Chen *et al*. (2010) with minor modifications [18]. Crude extracts from infected and healthy mulberry plants were diluted 1:30 and used as the antigen source and negative control, respectively.

### RNA extraction and rapid amplification of cDNA ends (RACE)

Total RNA was extracted from the infected mulberry leaves using RNAplant plus reagent (TIANGEN, Beijing, China) according to the manufacturer's instruction and the quality of the purified RNA was evaluated by gel electrophoresis on 0.8% (w/v) agarose gels. The 5′ and 3′-terminal sequences of the viral genomic RNA were determined by using the RACE cDNA Amplification Kit (BD Biosciences, Franklin Lakes, NJ, USA) coupling with Sanger sequencing. Primers were designed according to the available sequences obtained through viral small RNA deep sequencing. The RACE products were purified using DNA gel recovery kit (Sangon, Shanghai, China) and cloned into the pCR2.1-TOPO vector (Thermo Fisher Scientific, Waltham, MA, USA) before sequencing.

### Amplification and cloning of viral genomic cDNAs

MVBaV-specific primer pairs were designed based on the sequence information obtained by 5′ and 3′ RACE, and used for the amplification of complete fragments of the MVBaV genome. The primers LRNAF1/LRNAR1 and LRNAF2/LRNAR2 were used for the PCR amplification of MVBaV L RNA, MRNAF/MRNAR for MVBaV M RNA, and SRNAF/SRMAR for S RNA, respectively. The reverse transcription reaction was carried out using the Reverse Transcriptase Kit (Thermo Fisher Scientific) and the PCR was carried out in a total reaction volume of 25 μl. After denaturizing at 95°C for 2 min, the PCR were performed with the following parameters: 35 cycles with each cycle at 94°C for 30 s, 55–58°C for 30 s (the temperature varied with the length of template fragments), 72°C for 45 s, and final extension at 72°C for 10 min. The PCR products were analyzed by electrophoresis on a 0.8% (w/v) agarose gel. The PCR products of interest were recovered from the gel and cloned into pCR2.1-TOPO vector plasmid. The primers used for RACE and amplifying the MVBaV Genome are shown in Table 1.

**Table 1:** Primers used for amplification of the MVBaV genome.

Primer	Sequence (5' to 3')	Location and orientation
Tos-UPA	GACCACGCGTATCGATGTCGAC**AGAGCAATC**	3' termination of S, M and L RNA
SRNA 5–1	GCTGGGAACTGTGCTGTCAGAAAGGC	S 670-695(vc)
SRNA 5–2	ATGTTCTCTCTCCAGTAAGACGTTG	S 610-634(vc)
S RNA 3	ATTGCTCTCGCTCAACATCTTAAC	S 2547-2570(v)
S RNAF	AGAGCAATCAGGGTATTAATT	S 1-21(v)
S RNAR	AGAGCAATCGAGGTATTAATAAAAACA	S 3268-3294(vc)
MRNA 5–1	CACTTCTAGAGACTTGTCCATATC	M 961-984(vc)
MRNA 5–2	CTATATCAATGTCAATCTCAACCTC	M 937-961(vc)
M RNA 3	GCTATGCTTGATGGTATGTATGAAATAAG	M3378-3406(v)
MRNAF	AGAGCAATCGGTGCACGAATTCACAGAATC	M 1-30(v)
M RNAR	AGAGCAATCAGTGCAACAATTAAAGA	M4706-4731(vc)
LRNA 5–1	GGCCTGAATCAAGAATAGACAGAG	L1402-1425(vc)
LRNA 5–2	TCTTCTAGTGAGATAATGACCTCG	L 1241-1264(vc)
LRNA 3	TCAGTTATAGCATCTCTGAATGACTG	L 7380-7405(v)
LRNAF1	AGAGCAATCGAGCAACATATTAAAGTA	L 1-27(v)
LRNAR1	GCTACTATCCGACTTCAACTGC	L 4328-4349(vc)
LRNAF2	TTAGGGTTCAGGGTTATACAATAGC	L 4254-4278(v)
LRNAR2	AGAGCAATCGTGCAACAATAAAATATCAG	L 8877-8905(vc)

The cloned PCR products were sequenced with sequencer ABI 3700. At least three independent clones were sequenced for a target PCR product. The assembled full-length sequence data of MVBaV genome were deposited in the GenBank with accession numbers KM819698-KM819709.

### Analysis of viral RNA sequences

Sequence analysis was carried out using programs within the software VECTOR NTI V11.0 (Thermo Fisher Scientific). Phylogenetic trees were constructed by the neighbor-joining method with 1000 bootstrap replications using a program included in MEGA 6.06 [26]. The putative peptide cleavage sites and the *N*- and *O*-linked glycosylation sites of the GP of MVBaV were predicted using the software programs SignalP 4.1, NetNGlyc 1.0, and NetOGlyc 3.1, respectively. For transmembrane domain prediction, the TMHMM Server 2.0 program was used. The sequences of Tospoviruses were down loaded from the GenBank and listed in Table 2.

**Table 2:** List of accession number and abbreviation of Tospoviruses used in this study.

Virus	Abbreviation	S RNA	M RNA	L RNA
Mulberry vein banding associated virus	MVBaV	KM819701.1	KM819699.2	KM819698.1
Alstromeria necrotic streak virus	ANSV	GQ478668.1(N)	–	–
Bean necrotic mosaic virus	BeNMV	JN587268.1	JN587269.1	JF417980.1
Calla lily chlorotic spot virus	CCSV	AY867502.1	FJ822961.1	FJ822962.1
Capsicum chlorosis virus AIT isolate	CaCV-AIT	NC_008301.1	NC_008303.1	NC_008302.1
Capsicum chlorosis virus HT-1 isolate (synonyms: Gloxinia tospovirus)	CaCV-HT-1	AF059578 (N) AF059577(NSs)	AF023172.1	–
Chrysanthemum stem necrosis virus	CSNV	KM114548	KM114547	KM114546
*Groundnut bud necrosis virus*	GBNV	AY871098.2	U42555.1	AF025538.1
Groundnut chlorotic fan- spot virus	GCFSV	AF080526.1	–	–
*Groundnut ringspot virus* (South African isolate)	GRSV-SA	AF487516.1 for N, JN571117.1 for NSs	AY574055.2 for GPs, AF213673.1 for NSm	-
GRSV (south Florida isolate from the United State)	GRSV-US	HQ644140.1	HQ644141.1	HQ644142.1
*Groundnut yellow spot virus*	GYSV	AF013994.1	–	–
Hippeastrum chlorotic ringspot virus	HCRV	JX833564	JX833565	HG763861
*Impatiens necrotic spot virus*	INSV	X66972.1	DQ425095.1	DQ425094.1
Iris yellow spot virus	IYSV	AF001387.1	FJ361359.1	FJ623474.1
Lisianthus necrotic ringspot virus	LNRV	AB852525	–	–
Melon severe mosaic virus	MSMV	EU275149.1	–	–
Melon yellow spot virus	MYSV	AB038343.1	AB061773.1	AB061774.1
Pepper necrotic spot virus	PNSV	HE584762.1	–	–
Physalis silver mottle virus	PhySMV	AF067151	–	–
*Polygonum ringspot virus*	PolRSV	KJ541744	KJ541745	KJ541746
Soybean vein necrosis associated virus	SVNaV	HQ728387.1	HQ728386.1	HQ728385.1
*Tomato chlorotic spot virus*	TCSV	AF282982.1 (N)	AY574054.2 for GPs, AF213674.1 for NSm	HQ700667.1
Tomato necrosis virus	TNeV	AY647437 (N)	–	–
Tomato necrotic ringspot virus	TNRV	FJ489600.2	FJ947152.1	–
Tomato necrotic spot virus	TNSV	KM355773.1	–	–
*Tomato spotted wilt virus*	TSWV	AF020660.1	JN664253.1	NC_002052.1
Tomato yellow ring virus	TYRV	AY686718.1	JN560177	JN560178
Tomato zonate spot virus	TZSV	EF552433.1	EF552434.1	EF552435.1
Watermelon bud necrosis virus	WBNV	GU584184.1	GU584185.1	GU735408.1
*Watermelon silver mottle virus*	WSMoV	AB042650.1	DQ157768.1	AY863200.1
*Zucchini lethal chlorosis virus*	ZLCV	AF067069.1(N)	AB274027.1for GPs, AF213676.1 for NSm	–

## Results

### Symptomatology and virion morphology

**Fig 1:**
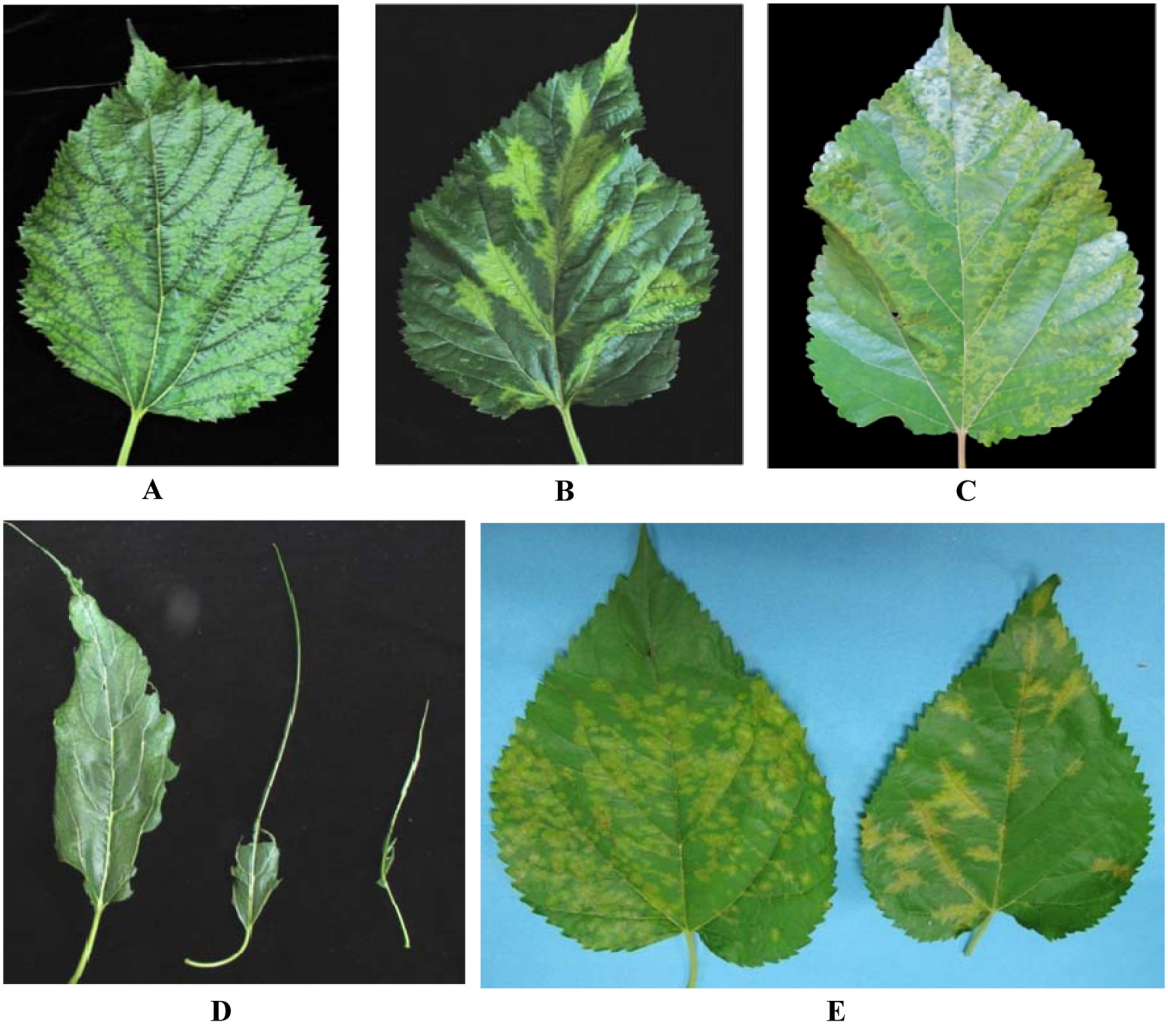
The symptoms of MVBaV-infected mulberry leaves. (A) vein banding, (B) mosaic, (C) chlorotic spots, (D) leaf deformation and (E) vein necrosis. The photos were taken over a period of eight months from April to November in 2013.

**Fig 2:**
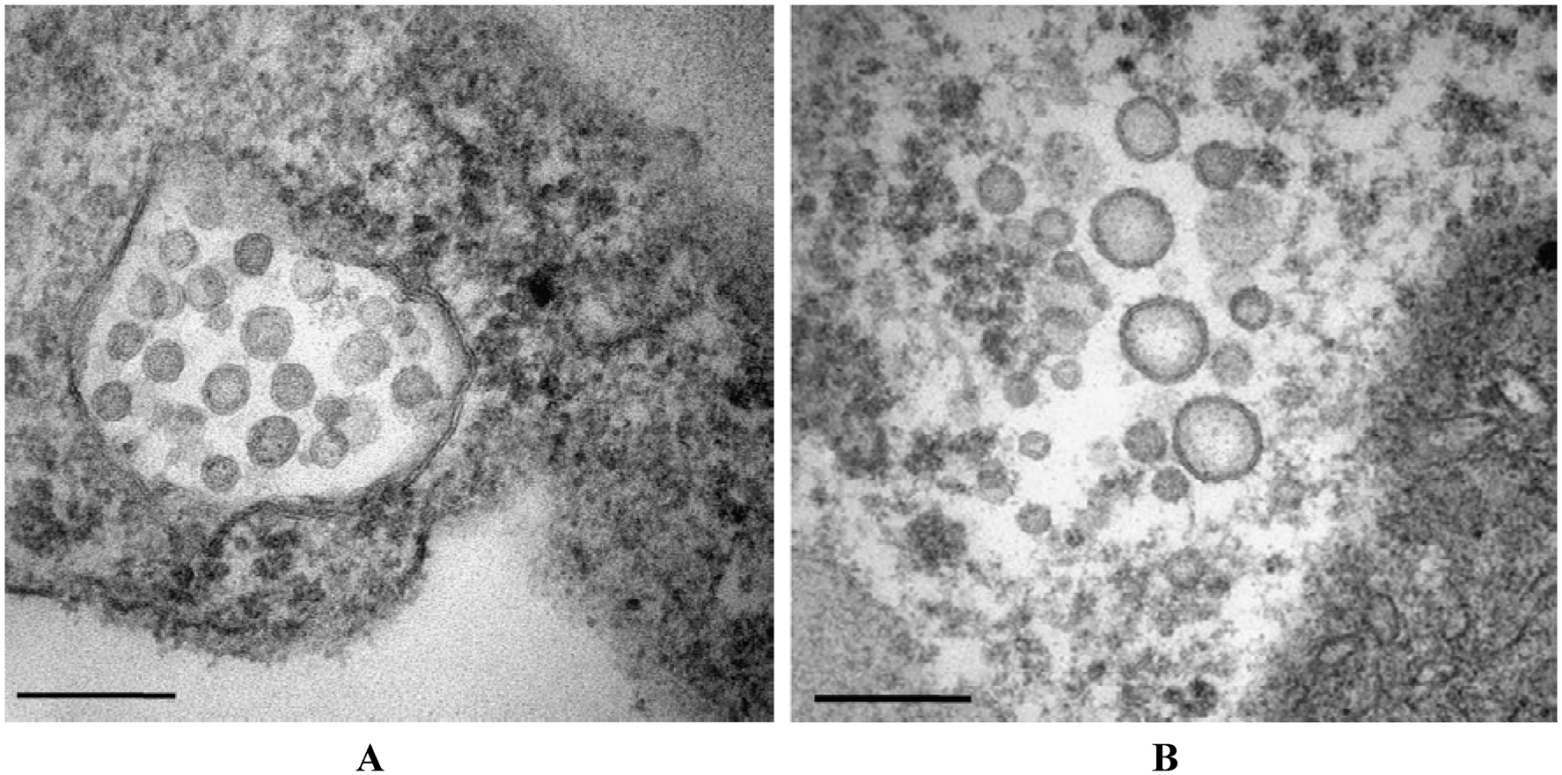
Electron micrograph of a MVBaV-infected mulberry plant leaves showing typical tospovirus-like particle morphology. Typical spherical, enveloped virions are shown accumulating in the endoplasmic reticulum (A) or dispersing in the cytoplasm as single particles in leaf cells (B). The scale bar represents 200 nm.

The symptoms of MVBaV-infected leaves varied widely, depending on the growing stage of mulberry and the environmental conditions. Initial symptoms of vein banding, mosaic, or chlorotic ringspots (Fig 1A–1C) were observed in early April and leaf deformation could develop later (Fig 1D). In the autumn, necrotic ringspots and vein necrosis on leaves were common (Fig 1E). The diseased mulberry leaf samples were further examined by electron microscopy. Enveloped particles with a quasi-spherical shape of 80–100 nm in diameter were observed to accumulate in the endoplasmic reticulum (Fig 2A) or dispersed in the cytoplasm as single particles (Fig 2B) in leaf cells. These particles had morphological features typical of tospoviruses. The tospovirus-like particles were also present in the symptom-showing leaves of plants infected by grafting inoculation (data not shown), suggesting that the symptoms were associated with MVBaV.

### Serological characterization of MVBaV

The diseased samples reacted strongly with WSMoV/GBNV mixed antisera, but not with antisera to TSWV, INSV, IYSV, and GRSV/TCSVin ELISA assays (data not shown), indicating that MVBaV is serologically related to the members of the WSMoV serogroup.

### Genome organization of the MVBaV

**Fig 3:**
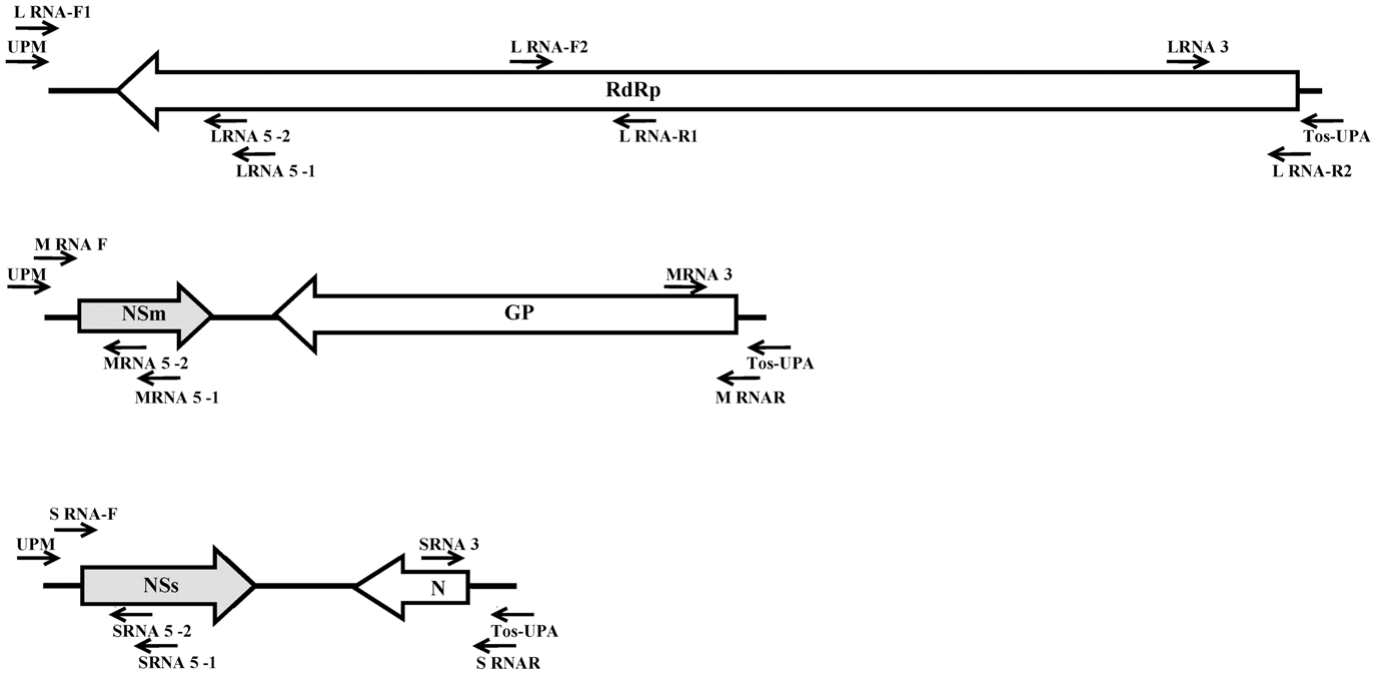
Cloning strategies for L, M, and S RNAs. Arrows indicate the annealing positions of each primer. To determine the 5′-terminal sequences, total RNAs were denatured by heating at 65°C for 5 min and then mixed with the first primer, LRNA 5–1 for L RNA, MRNA 5–1 for M RNA, and SRNA 5–1 for S RNA, respectively. After removal of template RNAs by RNaseH digestion, PCR amplification of the 5′-cDNAs was performed with Ex Taq DNA polymerase (Takara Bio, Dalian, China) using UPM primer (provided with the kits) and a nested primer, LRNA 5–2 for L RNA, MRNA 5–2 for M RNA, and SRNA 5–2 for S RNA, respectively. To determine the 3′-terminal sequence, first strand cDNA was synthesized by using the primer Tos-UPA. PCR amplification of the 3′-cDNAs was performed with Ex Taq DNA polymerase using the primers Tos-UPA and the LRNA 3 primer for L RNA, MRNA 3 for M RNA, and SRNA 3 for S RNA, respectively. The primers used for RACE and amplifying the MVBaV Genome are shown in Table 1.

In our previous deep sequencing of small RNAs from MVBaV-infected samples, three incomplete RNA segments of MVBaV responding to the L, M, and S RNA had been assembled (our unpublished data). The complete genome sequence of the MVBaV isolate XCSY-3 was obtained by combining the deep sequencing data, RT-PCR and RACE data (Fig 3, Table 1).

#### L RNA

Two overlapping PCR fragments generated by RT-PCR constitute the complete MVBaV L RNA of 8905 nt in length (GenBank accession number KM819698). There is a single open reading frame (ORF) in the viral complementary strand (vc-strand) that encodes a deduced RdRp (L protein) of 2877 aa with a molecular mass of 331.6 kDa. The 5' untranslatable regions (UTRs) and 3'-UTRs of the L RNA were 241 and 30 nt in length, respectively. The 17-nt termini of the L RNA formed a panhandle structure with a mismatch at the 11th nucleotide.

**Fig 4:**
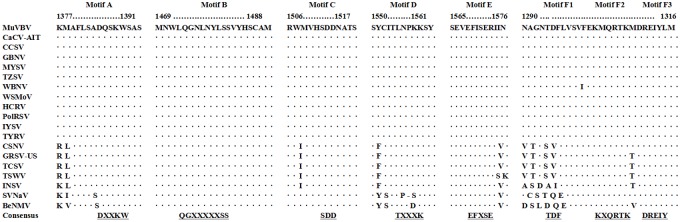
The RdRp conserved motifs in viruses of the genus *Tospovirus*. The consensus amino acid residues in each RdRp motif of the family *Bunyaviridae* are shown in bold and underlined. Identical amino acid (aa) residues are indicated with dots and deficient aa residues with hyphens. The positions of the conserved motifs in RdRp are indicated. Abbreviations and accession numbers of the analyzed sequences of tospoviruses are listed in Table 2.

The RdRp protein of MVBaV has a conserved region similar to those of other tospoviruses. The five conserved motifs, motif A (DXXKW), motif B (QGXXXXXSS), motif C (SDD), motif D (K), and motif E (EXXS), were all found within the RdRp of MVBaV. Furthermore, three motifs, Motif F1 (TDF), Motif F2 (KxQRTK) and Motif F3 (DREIY), which were found in the RdRp of CaCV, IYSV, TYRV, WBNV, GBNV, WSMoV, TZSV, and CCSV [21], were also present in the MVBaV RdRp (Fig 4).

#### M RNA

The complete nucleotide sequence of the MVBaV M RNA is 4731 nt in length and contains two ORFs encoding NSm and GP, in an ambisense coding strategy and separated by an IGR of 322 nts (GenBank accession number KM819699). The 5'- and 3'-UTRs of MVBaV M RNA contain 57 and 47 nts, respectively, forming a panhandle structure with 21 base pairs and 3 mismatches at the termini. The ORF on the viral strand codes for the NSm protein of 309 aa with a molecular mass of 34.3 kDa. The ORF on the vc-strand was 3,375 nt in length and codes for a 1,124 aa GP with a molecular mass of 128.1 kDa.

**Fig 5:**
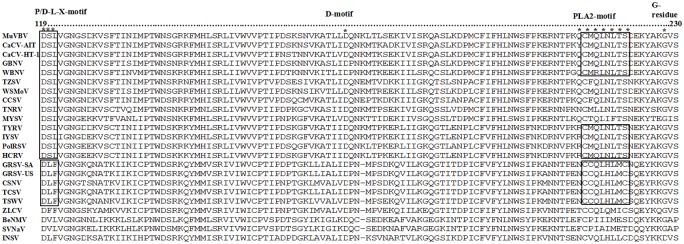
The NSm conserved motifs in viruses of the genus *Tospovirus*. The positions of the conserved motifs in NSm are indicated. Abbreviations and accession numbers of the analyzed sequences of tospoviruses are listed in Table 2.

The NSm protein, probably involved in cell-to-cell movement, belongs to the 30 K movement protein superfamily [27, 28]. The conserved motifs of the 30 K movement protein superfamily, such as the D-motif and G-residue, were found in the NSm protein of MVBaV, while the P/D-L-X motif and the phospholipase A2 catalytic site (PLA2-motif) present in NSm proteins of TSWV, GRSV, CSNV, and TCSV [28, 29], was absent from the MVBaV (Fig 5).

In topology of MVBaV GP protein, four *N*-glycosylation sites (N294, N549, N930, and N1101) and five transmembrane domains (aa positions 4–26, 297–316, 321–343, 414–436, and 1051–1073) were predicted and two potential signal peptides with predicted cleavage sites at aa 24 (VYL-LN) and aa 436 (SIA-LQ) to yield the glycoproteins Gn (47.8 kDa) and Gc (77.7 kDa) were present, whereas Arg-Gly-Asp (RGD) motif was not found.

#### S RNA

**Fig 6:**
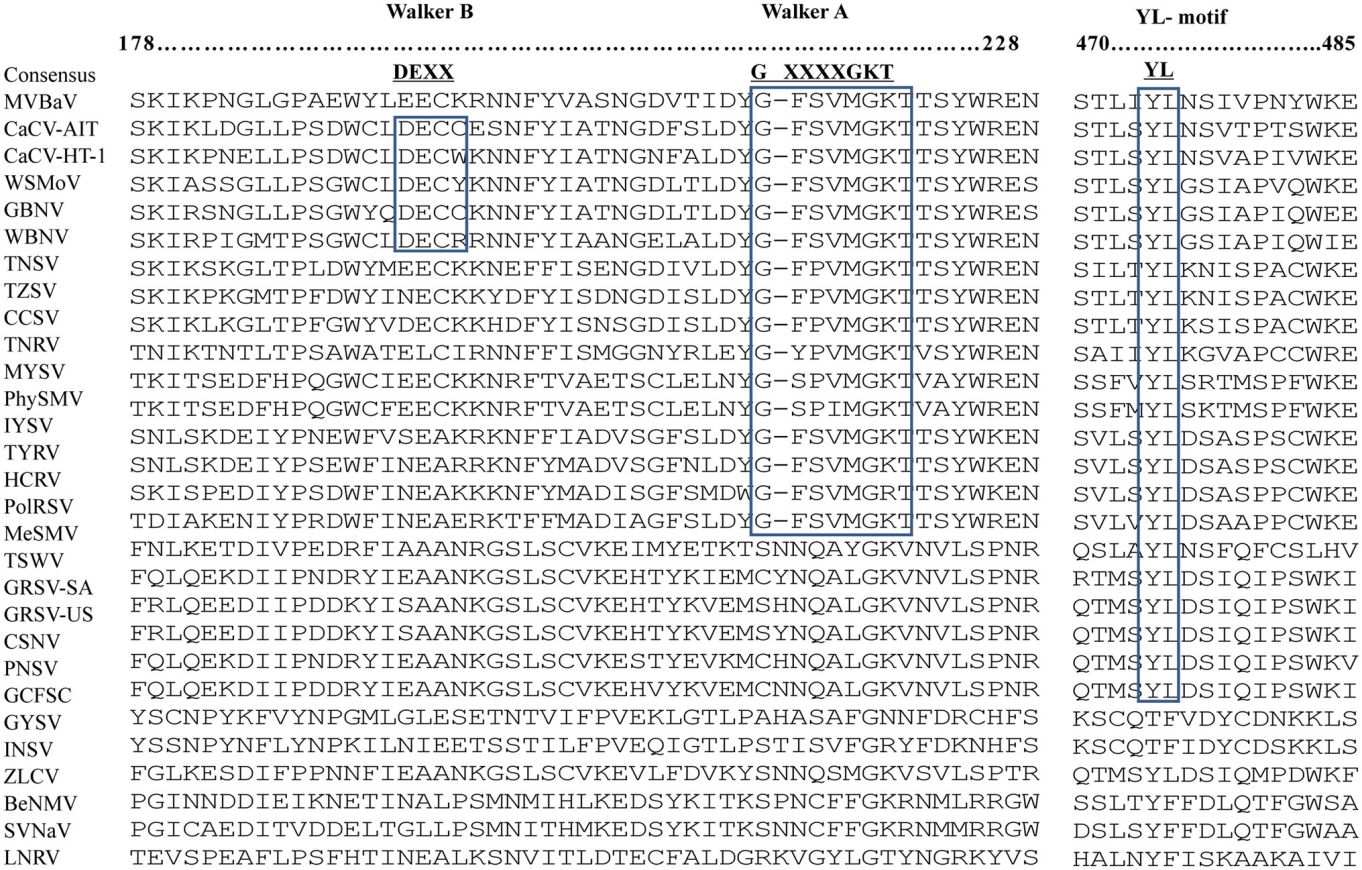
The NSs conserved motifs in viruses of the genus *Tospovirus*. The positions of the conserved motifs in NSs are indicated. Abbreviations and accession numbers of the analyzed sequences of tospoviruses are listed in Table 2.

The complete nucleotide sequence of the MVBaV S RNA is 3294 nt in length (GenBank accession code KM819701). The S RNA contains two ORFs in an ambisense coding strategy separated by an AU-rich IGR of 1008 nt in length spun from coordinates 1386–2393 in the v-strand. The 65- and 67-nt untranslated regions in the 5'- and 3'-UTRs can form a panhandle structure of 22 bp with 2 mismatches. The ORF on the v-strand codes for a 439 aa NSs protein with a molecular mass of 49.1 kDa and the ORF on the vc-strand encodes a 277 aa nucleoprotein (N protein) of 30.69 kDa. Three highly conserved motifs, Walker A (GxxxxGKT), Walker B (DEXX), and YL, were present in the NSs proteins of some tospoviruese [30, 31]. Walker A and YL were present (aa positions 175–182 and 416–417), while the Walker B motif was not found in the NSs protein of MVBaV (Fig 6).

### Evolutionary relationship of the MVBaV to other tospoviruses

MVBaV RdRp shares the highest sequence homology to that of WSMoV (85.8%) and high sequence homology to GBNV, WBNV, and CaCV (83.9–84.9%). To the rest members of the WSMoV serogroup (MYSV, TZSV, CCSV), the homology were 76.1–77.8%. Intriguingly, the RdRp homology between MVBaV and topoviruses of different serotypes was all less than 70%, with members of TSWV and the distinct serotypes being the lowest (42.3–45.2%) (Table 3). An inspection of the proteins encoded by the M RNA and S RNA of different tospoviruses revealed that MVBaV was closest to WSMoV serogroup (M RNA, 64.6–83.2% homology; S RNA, 45.4–74.4% homology) and most distant from TSWV, GYSV, and Distinct (M RNA, 34.1–39.2% homology; S RNA, 15.9–29.9% homology), with IYSV serogroup in between (M RNA, 66.2–68.5% homology; S RNA, 41.9–49.4% homology).

**Table 3:** Comparison of the RNAs and deduced proteins of MVBaV with those of other Tospoviruses.

M RNA	Full length	5'UTR	NSm			IGR	GPs			3'UTR
	nt	nt	nt	aa	Identity (%)	nt	nt	aa	Identity %	nt
**WSMoV serogroup**										
MVBaV	8905	241	8634	2877		30				
WSMoV	8917	248	8637	2878	85.8	32				
GBNV	8911	245	8634	2877	84.9	32				
WBNV	8916	247	8637	2878	84.6	32				
CaCV-AIT	8912	247	8634	2877	84.5	31				
CCSV	8911	230	8652	2883	77.8	29				
TZSV	8919	233	8658	2885	76.9	28				
MYSV	8918	273	8613	2870	76.1	32				
**IYSV serogroup**										
HCRV	8908	250	8622	2873	69.0	33				
PolRSV	8893	230	8631	2876	68.8	32				
TYRV	8877	223	8622	2873	68.5	32				
IYSV	8880	225	8622	2873	67.8	33				
**TSWV serogroup**										
CSNV	8955	397	8625	2874	45.2	33				
GRSV-US	8876	217	8625	2874	45.2	34				
TCSV	8868	215	8622	2873	44.8	31				
TSWV	8897	236	8628	2875	43.1	33				
**Other serotype**										
INSV	8780	147	8598	2865	44.9	35				
SVNaV	9010	184	8796	2931	42.5	30				
BeNMV	9040	220	8799	2932	42.3	21				
**M RNA**	Full length	5'UTR	NSm			IGR	GPs			3'UTR
	nt	nt	nt	aa	Identity (%)	nt	nt	aa	Identity (%)	nt
**WSMoV serogroup**										
MVBaV	4731	57	930	309	—	322	3375	1124	—	47
CaCV-AIT	4823	56	927	308	82.6	427	3366	1121	80.9	47
CaCV-HT-1	4780	29	927	308	82.3	436	3369	1122	78.9	19
GBNV	4801	56	924	307	81.9	408	3366	1121	84.3	47
WBNV	4794	55	924	307	79.9	402	3366	1121	81.6	47
TZSV	4945	54	930	309	77.3	546	3369	1122	73.2	46
WSMoV	4877	55	939	312	75.2	470	3366	1121	80.7	47
CCSV	4704	54	930	309	75.1	303	3372	1123	73.6	45
TNRV	4716	59	933	310	73.1	307	3369	1122	65.3	48
MYSV	4815	58	927	308	64.6	398	3384	1127	63.4	48
**IYSV serogroup**										
TYRV	4786	62	927	308	68.5	354	3393	1130	62.9	50
IYSV	4821	63	936	311	68.1	394	3411	1136	60.3	17
PolRSV	4710	62	927	308	66.2	263	3408	1135	60.0	50
HCRV	4741	47	927	308	65.9	330	3390	1129	61.9	47
**TSWV serogroup**										
GRSV-SA	—	—	912	303	39.2	—	3402	1133	33.4	—
GRSV-US	4848	99	912	303	38.6	348	3405	1334	34.1	84
CSNV	4830	101	912	303	38.6	326	3408	1135	34.5	83
TCSV	—	—	912	303	38.6	—	3405	1134	34.1	—
TSWV	4767	100	909	302	37.5	266	3408	1135	34.1	84
ZLCV	—	—	912	303	34.3	—	3408	1135	34.5	—
**Other serotype**										
BeNMV	4886	64	954	317	37.6	299	3486	1161	33.1	83
INSV	4962	85	912	303	36.9	465	3333	1110	32.9	86
SVNaV	4955	57	951	316	34.1	267	3588	1195	30.1	92
**S RNA**	Full length	5'UTR	NSs			IGR	N			3'UTR
	nt	nt	nt	aa	Identity (%)	nt	nt	aa	Identity (%)	nt
**WSMoV serogroup**										
MVBaV	3294	65	1320	439		1008	834	277		67
CaCV-AIT	3477	66	1320	439	71.3	1199	828	275	74.4	67
CaCV-HT-1	—	—	1320	439	69.5	—	834	277	72.9	—
TNeV	—	—	—	—	—	—	828	275	74.0	—
WSMoV	3558	67	1320	439	70.8	1278	828	275	70.0	65
GBNV	3057	66	1320	439	70.6	773	831	276	71.5	67
WBNV	3401	66	1320	439	70.4	1120	828	275	71.8	67
TNSV	3012	66	1380	459	61.7	664	834	277	61.5	68
TZSV	3279	64	1380	459	60.6	934	837	278	59.4	64
CCSV	3172	66	1383	460	59.6	825	834	277	60.4	64
TNRV	3023	65	1356	451	47.3	690	846	281	56.2	66
MYSV	3232	68	1410	469	45.4	847	840	279	58.4	67
PhySMV	3257	68	1410	469	45.0	872	840	279	58.4	67
**IYSV serogroup**										
IYSV	3105	70	1332	443	49.4	811	822	273	44.8	70
TYRV	3061	71	1332	443	49.4	762	825	274	44.1	71
HCRV	2744	73	1338	445	48.1	437	825	274	42.7	71
PolRSV	2485	73	1332	443	46.7	183	825	274	41.9	72
**TSWV serogroup**										
MeSMV	3283	80	1368	455	20.2	887	789	262	28.5	159
TSWV	2955	88	1404	467	17.7	535	777	258	29.9	151
GRSV-SA	—	—	1404	467	17.5	—	—	235	28.5	—
GRSV-US	3049	87	1404	467	17.7	630	777	258	29.2	151
CSNV	2947	79	1404	467	17.3	529	783	260	27.0	152
PNSV	2949	87	1404	467	17.0	529	777	258	28.5	153
ZLCV	—	—	—	—	—	—	783	260	28.5	—
TCSV	—	—	—	—	—	—	777	258	28.1	—
ANSV	—	—	—	—	—	—	777	258	28.1	—
**GYSV serotype**										
GCFSV	2833	67	1419	472	16.3	455	813	270	20.8	79
GYSV	2970	57	1443	480	15.9	653	741	246	22.9	76
**Other serotype**										
INSV	2992	62	1350	449	19.9	642	789	262	24.3	149
BeNMV	2584	60	1320	439	19.1	315	813	270	27.3	76
SVNaV	2603	58	1323	440	18.8	318	834	277	29.8	70
LNRV	2768	85	1326	441	17.7	385	807	268	19.9	159

Of particular interest is the envelop glycoproteins (GPs). Although the aa homology between MVBaV and most members of WSMoV serogroup is greater than 70%, two members, TNRV and MYSV, share only 63.4% and 65.3% homology, compatible to the 62.9% of TYRV, a member belonged to IYSV serogroup.

**Fig 7:**
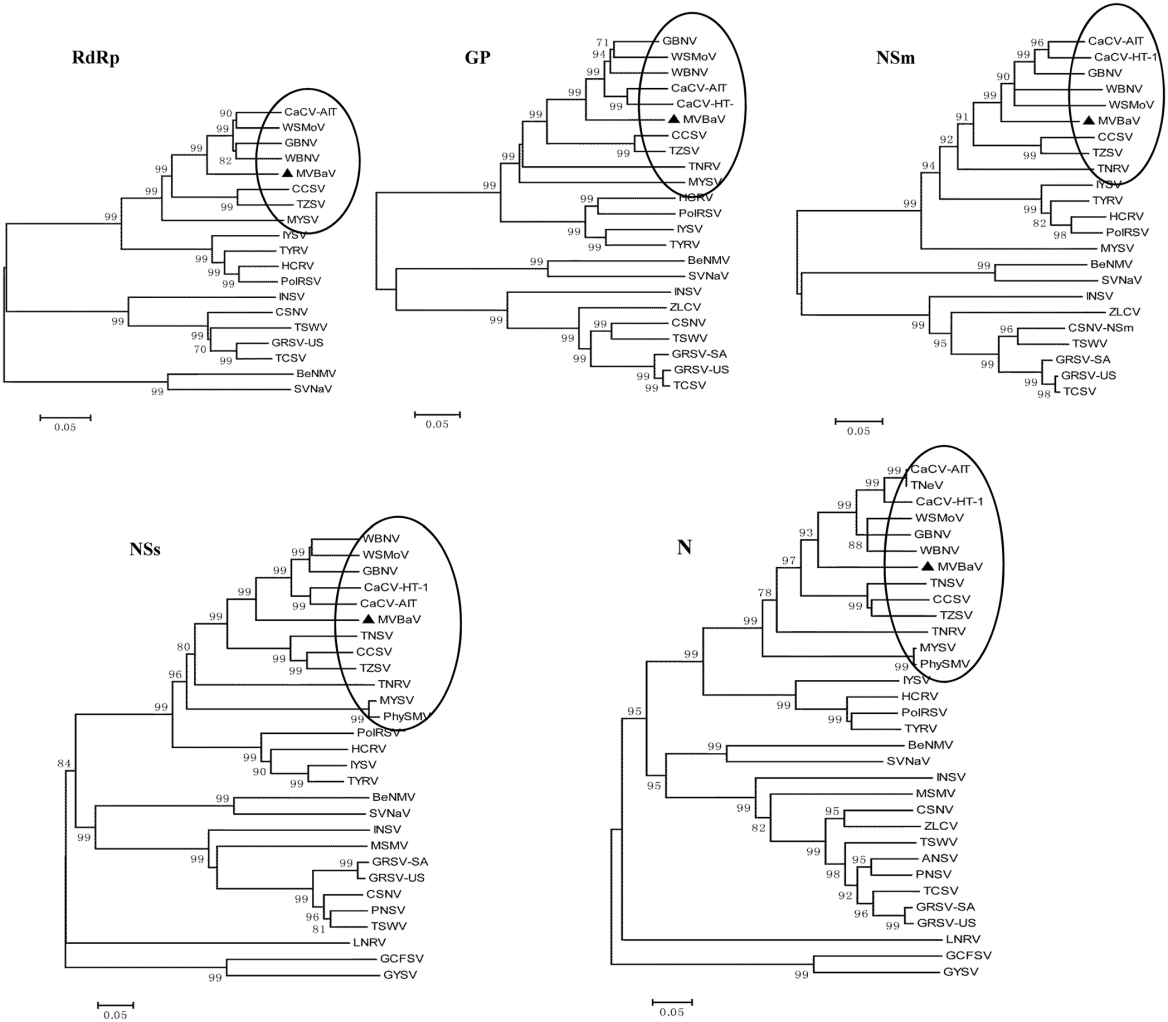
Phylogenetic trees of tospoviruses. Amino acid sequences of the RNA-dependent RNA Polymerase (RdRp), GP protein (GP),NSm protein (NSm),NSs protein (NSs), and N protein (N) were used to constructed the trees. The dendrograms were produced using the Neighbour—Joining algorithm with 1000 bootstrap replicates. The tospovirus species belonging to WSMoV serogroup are circled. The scale bar represents a relative genetic distance. Numbers above critical branches are significant bootstrap values (>70%). Abbreviations and accession numbers of the analyzed sequences of tospoviruses are listed in Table 2.

The phylogenetic trees constructed with RdRps, NSm, GnG, NSs, and N all clearly showed that MVBaV was a distict member of WSMoV serogroup, more closely related to a subgroup composed of CaCV, WBNV, WSMoV, and GBNV (Fig 7).

### S RNA sequence diversity

To assess the genome sequence diversity of MVBaV, the S RNA of nine additional MVBaV isolates were cloned, sequenced, and compared with that of the type isolate XCSY-3. As shown in Table 3, the lengths of the S RNA of these isolates range from 3273 to 3324 nt with six length patterns and the IGRs from 987 to1038 nt with 7 patterns among a total of 10 isolates. However, the 5'-UTR and 3'-UTR were completely conserved at length of 65 and 67 nt, respectively. The N and NSs proteins were highly conserved with only one aa substitution in the 277-aa N protein in two isolates and 2–6 aa substitutions in the 439-aa NSs proteins in eight isolates (Table 4). Therefore, the rich sequence diversity of the S RNA was reflected mainly in the length of the RNA fragment and the IGS.

**Table 4:** Comparison of the S RNA from isolate XCSY-3 to those of nine additional MVBaV isolates.

Isolates (Accession number)	Full length	5'UTR	NSs			IGR	N			3'UTR
	nt	nt	Nt	aa	Identity (%)	nt	nt	aa	Identity (%)	nt
XCSY-3 (KM819701)	3294	65	1320	439		1008	834	277	-	67
XCBY-1 (KM819709)	3273	65	1320	439	99.5	987	834	277	100	67
XZDL-1 (KM819704)	3293	65	1320	439	99.3	1007	834	277	99.6	67
SL-3 (KM819700)	3324	65	1320	439	99.3	1038	834	277	100	67
HX-2 (KM819702)	3294	65	1320	439	100	1008	834	277	99.6	67
NN-5 (KM819705)	3288	65	1320	439	99.1	1002	834	277	100	67
NN-10 (KM819706)	3288	65	1320	439	98.4	1002	834	277	100	67
NN-16 (KM819708)	3276	65	1320	439	99.3	990	834	277	100	67
YZ-3 (KM819703)	3293	65	1320	439	98.6	1007	834	277	100	67
YZ- 4(KM819707)	3286	65	1320	439	99.8	1000	834	277	100	67

## Discussion

The N protein sequence, in combination with biological characters such as the thrips vector species and host range, represents the main classification criteria for the establishment of a new tospovirus species. In term of molecular differentiation, N protein sequence identity of 90% has been set as the threshold to establish a new species of tospovirus. As of to date, nine definitive species and fourteen tentative species of tospoviruses have been recognized by the International Committee on Taxonomy of Viruses [7, 32]. Among the tentative species, Iris yellow spot virus and Watermelon bud necrosis virus are pending for final approval as definitive species [32]. The recently reported vain-banding syndrome on mulberry (Fig 1) added a new plant to the host list of tospovirus [3]. Data of genome analysis presented in this study clearly demonstrates that MVBaV is indeed a new member of *Tospovirus*.

The genome of MVBaV is composed of L, M, and S RNAs. The genome organization strategy and the size as well as the proteins encoded are typical of the genus *Tospovirus*, particularly the WSMoV serogroup (Table 3). Phylogenetic analyses with five functional proteins encoded in L, M, and S RNAs all show that MVBaV is a distinct member of the WSMoV serogroup (Fig 7). This conclusion was also supported by the serological investigation. RdRp is responsible for the genome replication and may associates with host factors to fulfill its function [4]. Alignment of RdRp aa sequences revealed that all six conserved motifs for tosopoviruses were present in the RdRp of MVBaV (Fig 4). The highest homology of viral proteins between MVBaV and other topoviruses was found in RdRp, 83.9–85.8% to that of WSMoV, GBNV, WBNV, and CaCV. These five species form a subgroup within the WSMoV serogroup, while the rest members in the same serogroup share homology of 76.1–77.8% with MVBaV, compared with 67.8–69% for viruses from the IYSV serogroup and 42.5–45.2% for viruses from TSWV and distinct serotypes (Table 3). High homology in RdRp may suggest a conserved functional mechanism for this protein.

The NSm protein encoded by M RNA is thought to be probably involved in cell-to-cell movement and belongs to the 30 K movement protein superfamily [27, 28]. Two of the four feature motifs, P/D-L-X motif and PLA2-motif, characteristic in TSWV serotype viruses did not found their counterparts in viruses of other seropgroups. For P/D-L-X motif, rather than DLF, all other tospovurses except BeNMV and SVNaV, have a motif of DSL; for PLA2-motif, instead of CCQLHLMC, most WSMoV serogroup viruses contain CMQLNLTS, following the motif pattern CXXLNLTS. Of particular note is that MYSV contains a sequence of CTQLIFTS in this motif positions, very different from all other members in the WSMoV serogroup (Fig 5).

Tospoviruses are transmitted by insects belonging to the order Thysanoptera [33]. Glycoproteins encoded by the M RNA had been shown to be involved in vector transmission in TSWV virus [34, 35]. The glycoproteins (GPs) of MVBaV are 84.3% homologous to GBMV and 78.7–81.6% to CaCV, WSMoV, and WBNV (Table 3). Similarity in glycoproteins may imply a similar insect vector. Members of WSMoV serogroup all have apparent origin in Asia and are predominantly transmitted by only two species of the genus *Thrips* that have been proposed as WSMoV-Thrips-Asian type[4]. *Thrips palmi*, the major vector of the several tospoviruses in the WSMoV serogroup, was found abundantly in agriculture fields in China [36, 37]. Since *Pseudodendrothrips mori* is widely distributed in the mulberry field in China [38], it may well be the vector for MVBaV. Confirmation of the disease transmissibility by this thrips is currently under investigation.

N proteins encoded by the S RNA contain antigenic determinants that form the bases for the serotype differentiation in tospoviruses [18, 19]. The N protein of MVBaV shares the highest homology of 74.4% with that of CaCV, 70.0–71.8% with those of WSMoV, GBNV, and WBNV, and 56.2–61.5% to the rest of members in the WSMoV serotype (Table 3). It was proposed that tospovirus species sharing N homology identity above 51.8% were serologically related [39].

The NSs protein encoded in S RNA has been shown to participate in viral movement, replication, and suppression of the host defense mechanism in TSWV and GBNV [30, 40, 41]. The NSs protein of MVBaV shares 70.2–71.3% homology with CaCV, WSMoV, GBNV, and WBNV, 54.4–61.7% with the rest members of the WSMoV serogroup. This protein also shares homology of 46.7–49.6% with members of the IYSV serogroup, but only 17.7% homology with TSWV (Table 3). Alignment of NSs proteins showed that WSMoV has the typical Walker A and YL motifs, but not the Walker B motif (Fig 6).

Phylogenetic analysis and homology calculation of the viral proteins RdRp, NSm, GP, NSs, and N of tospoviruses places MVBaV between the CaCV subgroup composed of CaCV, GBNV, WSMoV, and the CCSV subgroup composed of CCSV, TZSV, TRNV, and MYSV within the WSMoV serogroup of *Tospovirus* (Fig 7, Table 3). Thus MVBaV may represent a species that bridges the lineage within the WSMoV serogroup of *Tospovirus*.

Genetic diversity of MVBaV was investigated by sequencing the S RNA of 10 MVBaV isolates collected from mulberry orchards in different locations in this study. As shown in Table 4, the sizes of the RNA range from 3273 to 3324 nt, showing a disperse pattern of 3288 (2 isolates), 3293 (2 isolates), 3294 (2 isolates), 3273 (1 isolate), 3276 (1 isolate), 3286 (1 isolate), and 3324 nt (1 isolate). However, the 5’ UTR (65 nt), 3’ UTR (67 nt), the NSs (1320 nt, 439 aa), and N (834 nt, 277 aa) are completely conserved in length among all isolates. Minor substitutions in NSs (2–6 in 439 aa, 9 isolates) and N (1 in 277 aa, 2 isolates) were observed. Variations in size of the S RNA were caused by the varied length in the IGR, which ranges from 987 to 1038 nt. Intriguingly, the IGR length pattern was perfectly matched with that of S RNA size pattern, i.e., S RNA size of 3288 nt with IGR of 1002 nt (isolates NN-5 and NN-10); S RNA size of 3293 nt with IGR of 1007 nt (isolates YZ-3 and XZDL-1); S RNA size of 3294 nt with IGR of 1008 nt (isolates XCSY-3 and HX-2). Thus, it is suggested that IGR of S RNA can be used as a genetic marker for MVBaV population analysis.

Genome reassortment and recombination play a role in the evolution of RNA virus. In this regard, genome reassortment and recombination have been observed to take place in several species of *Tospovirus* [42–43]. It was noted that the proteins GPs and NSm encoded by the M RNA of GRSV-US, a strain from the south Florida of the United State [43], do not group with the South African isolate (GRSV-SA), but with those of TCSV (Fig 7), demonstrating a genome fragment reassortment. Indeed, a large survey of the GRSV in vegetables in Florida and the Southeastern United States showed that all GRSV contained a reassorted genome with M RNA from TCSV [44]. Although no genome reassortment event has been observed for any of the ten *Tospovirus* members reported in mainland China [6, 12, 13, 45, 46], detailed survey by screening the genome components is required to evaluate the epidemic potential of these viruses, as genome reassortment may change the virus-vector or virus—host specificity.

We did not succeed in transmitting MVBaV by sap inoculation to any plants including mulberry. However, the virus could be transmitted by grafting (data not shown), establishing a positive correlation of MVBaV to the disease. Of important note, 32 out of 48 (66.7%) mulberry plants with viral disease-like symptoms were found to be infected with MVBaV in our preliminary survey, demonstrating the dominant nature of MVBaV in the field [3].The high incidence of MVBaV in the mulberry orchards and the vast importance of tospovirus to crops in general imply that this virus may represent a substantial threat to the silkworm industry in China [3]. Future studies should focus on the biology of MVBaV, including the plant-virus interaction and vector-virus interaction, disease epidemiology, and practical measures for the disease control. In these regards, elucidation of the genome organization of MVBaV lays a solid foundation for future studies.

## Supporting Information

S1 Table Information of virus sampling, virus-induced symptom and permission for collection of the samples of Mulberry vein banding associated virus (MVBaV). (DOC)
