# Supplementary material for: Complete Genome Sequence of Mulberry Vein Banding Associated Virus, a New Tospovirus Infecting Mulberry
Source: PLoS One. 2015 Aug 20;10(8):e0136196. doi: 10.1371/journal.pone.0136196 (PMC4546196; doi:10.1371/journal.pone.0136196)
Supplement: S1 Table — (DOC) [file pone.0136196.s001.doc]

**Complete genome sequence of Mulberry vein banding associated virus, a new *Tospovirus* infecting mulberry**

**Jiao-Rong Meng^1,2^, Ping-Ping Liu^1^, Li-Ling Zhu^1^, Cheng-Wu Zou^1^, Jie-Qiu Li^1^, Bao-Shan Chen^2*^**

**1** College of Agriculture, Guangxi University, Nanning, China, 2 State Key Laboratory for Conservation and Utilization of Subtropical Agro-bioresources (Guangxi University) and Key Laboratory of Ministry of Education of China for Microbial and Plant Genetic Engineering, Nanning, China

***** **E-mail: chenyaoj@gxu.edu.cn**

**S1 Table. Information of virus sampling, virus-induced symptom and permission for collection of the samples of Mulberry vein banding associated virus (MVBaV)**

| **No.** | **Sample Name** | **Sampling locations** | **Sampling time** | **Symptoms** | **Permit affiliation** | **Address** |
| --- | --- | --- | --- | --- | --- | --- |
|  | XCSY-3 | Xincheng County | 2011-09-11 | mosaic | [Agricultural](javascript:void(0);) [Bureau](javascript:void(0);) of Xincheng County | Chengguan Town, Xincheng County, Guangxi Province 546200, China |
|  | XCBY-1 | Xincheng County | 2011-04-5 | vein banding | [Agricultural](javascript:void(0);) [Bureau](javascript:void(0);) of Xincheng County | Chengguan Town, Xincheng County, Guangxi Province 546200, China |
|  | XZDL-1 | Xiangzhou County | 2012-04-08 | mosaic | [Agricultural](javascript:void(0);) [Bureau](javascript:void(0);) of Xiangzhou County | No. 130 Wenquan Road, Xiangzhou County, Guangxi Province 545800 , China |
|  | SL-3 | Shanglin County | 2011-09-05 | necrotic ringspots | [Agricultural](javascript:void(0);) [Bureau](javascript:void(0);) of Xincheng County | No.2 Yinquan Road, Shanglin County Guangxi Province, 530500, China |
|  | HX-2 | Heng County | 2012-04-24 | vein banding | [Agricultural](javascript:void(0);) [Bureau](javascript:void(0);) of Heng County | Hengzhou Town, Heng County, Guangxi Province 530300, China |
|  | NN-5 | Nannning City (GXU) | 2012-03-29 | mosaic | Guangxi University | Guangxi University, Nannning City, Guangxi Province 530005, China |
|  | NN-16 | Nannning City (GXU) | 2011-12-16 | vein necrosis | Guangxi University | Guangxi University, Nannning City, Guangxi Province 530005, China |
|  | NN-10 | Nannning City (GSTES) | 2012-04-17 | chlorotic ringspots | Guangxi Sericulture Technology Extension Station | Guangxi Sericulture Technology Extension Station , Nannning City, Guangxi Province 530007, China |
|  | YZ-3 | Yizhou City | 2012-04-21 | mosaic | [Agricultural](javascript:void(0);) [Bureau](javascript:void(0);) of Yizhou City | Qingyuan Town, Yizhou City, Guangxi Province 546300, China |
|  | YZ-4 | Yizhou City | 2012-04-21 | necrotic ringspots | [Agricultural](javascript:void(0);) [Bureau](javascript:void(0);) of Yizhou City | Qingyuan Town, Yizhou City, Guangxi Province 546300, China |

GXU: Guangxi University

GSTES: Guangxi Sericulture Technology Extension Station
